# Supplementary figures and images for: Alzheimer’s risk factor FERMT2 promotes the progression of colorectal carcinoma via Wnt/β-catenin signaling pathway and contributes to the negative correlation between Alzheimer and cancer
Source: PLoS One. 2022 Dec 8;17(12):e0278774. doi: 10.1371/journal.pone.0278774 (PMC9731493; doi:10.1371/journal.pone.0278774)

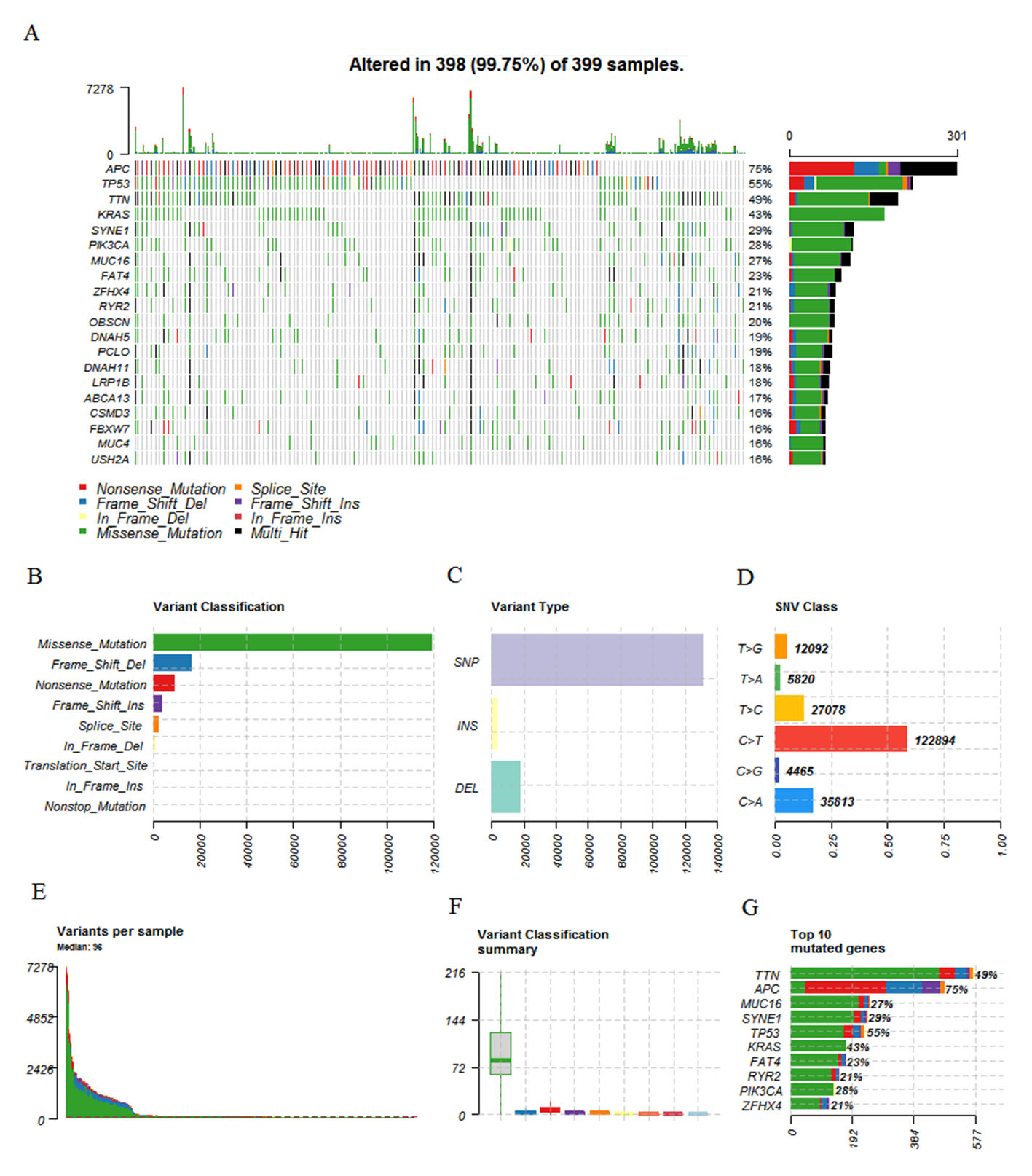

Supplement: S1 Fig — (A) Waterfall plot of detailed mutation information of top 20 genes in each sample, with various color annotations to distinguish different mutation types. (B-D) According to different classification categories, missense mutation, SNP, and C > T mutation accounted for the overwhelming majority. (E) The total mutation number in each sample. (F) Box plots of each variant classification in each sample. (G) Top 10 mutated genes in CRC. SNP, single nucleotide polymorphism; SNV, single nucleotide variants. (TIF) [file pone.0278774.s001.tif]

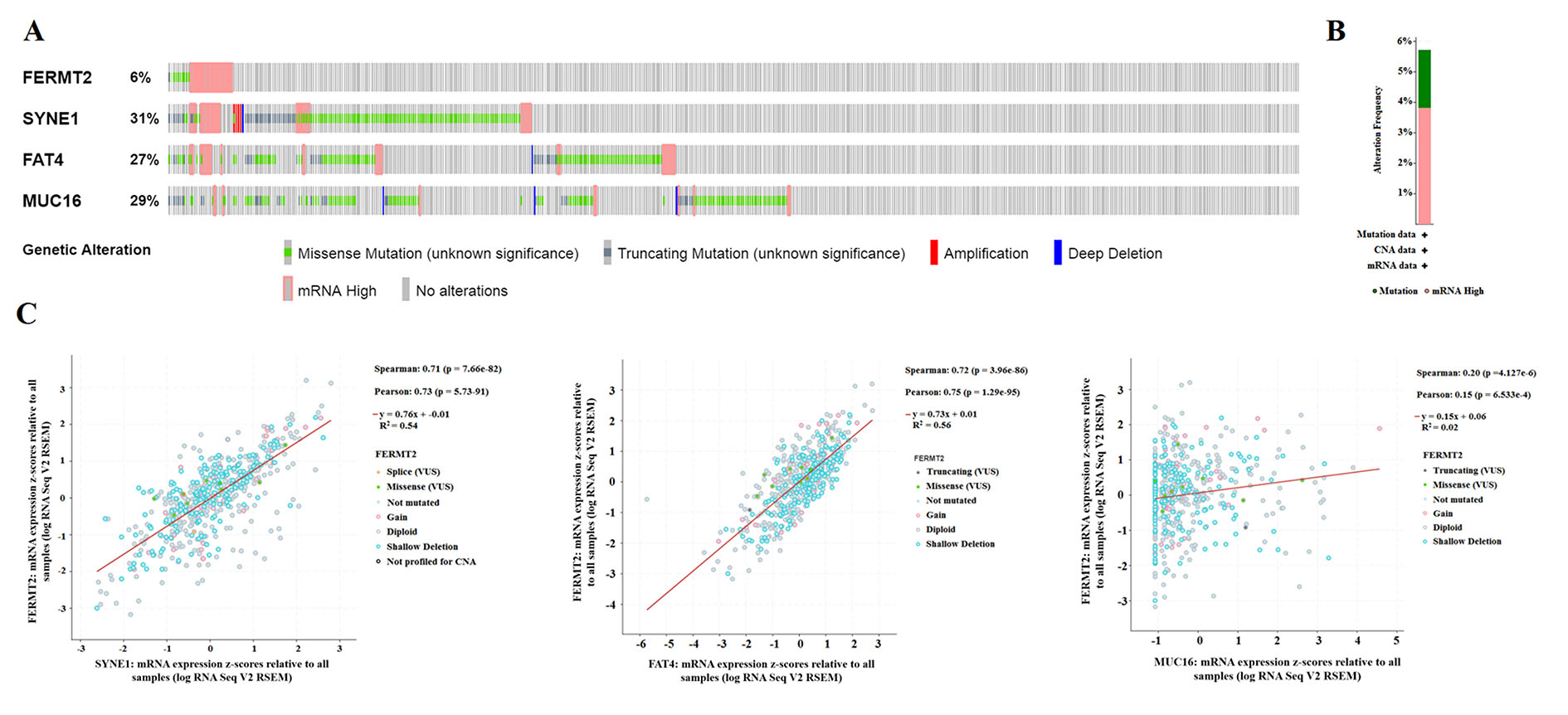

Supplement: S2 Fig — (A, B) OncoPrint of FERMT2 co-occurring alterations in CRC cohort. The different types of genetic alterations are highlighted in different colors. (C) Correlations between FERMT2 with SYNE1, FAT4 or MUC16 at mRNA expression levels in patients with CRC. (TIF) [file pone.0278774.s002.tif]

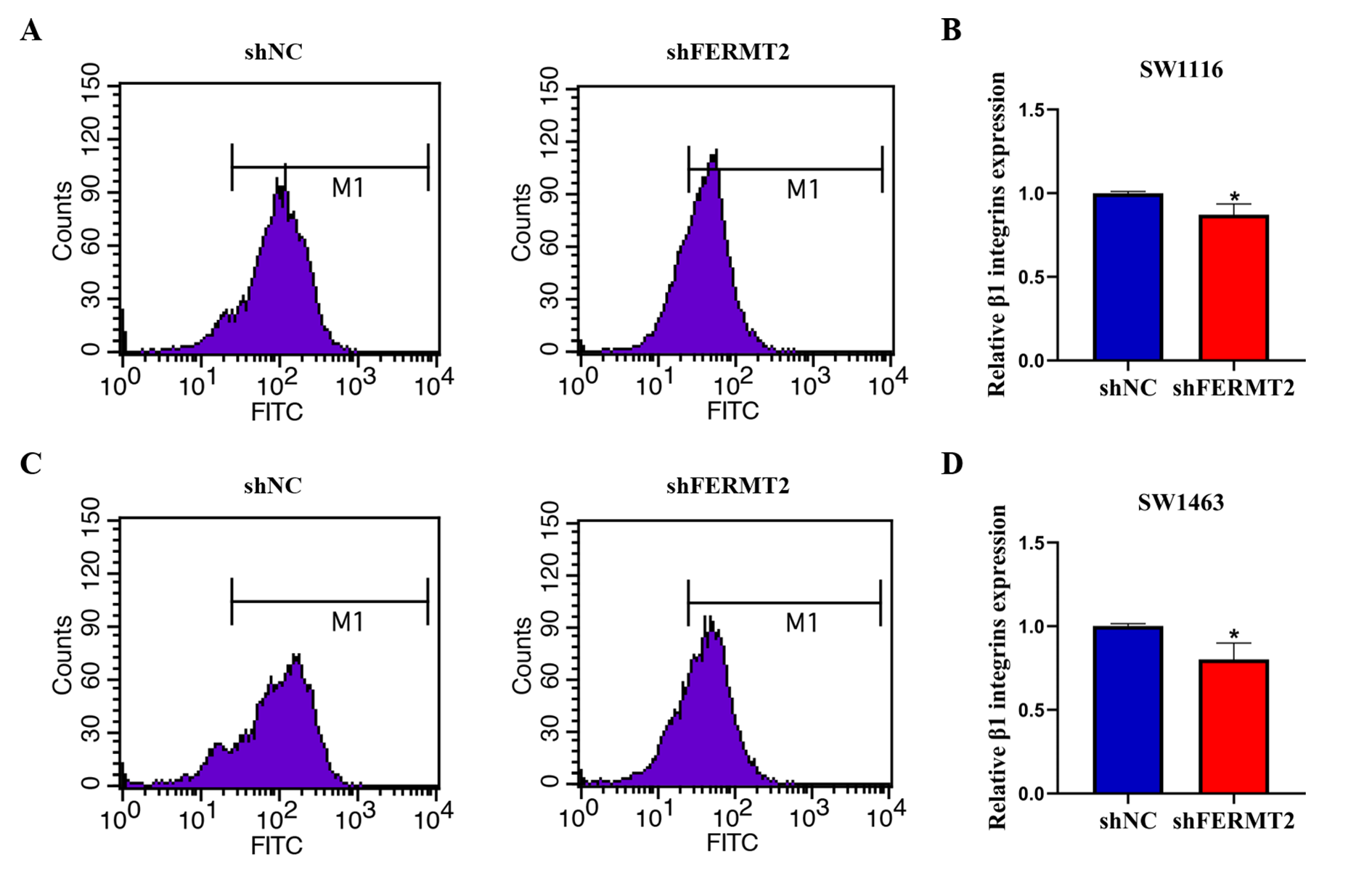

Supplement: S3 Fig — (A-D) The expression of β1 integrins in SW1116 cells (A, B) or SW1463 cells (C, D) after FERMT2 knockdown was measured by FACS. *P < 0.05 vs shNC. (TIF) [file pone.0278774.s003.tif]

Fig 5B

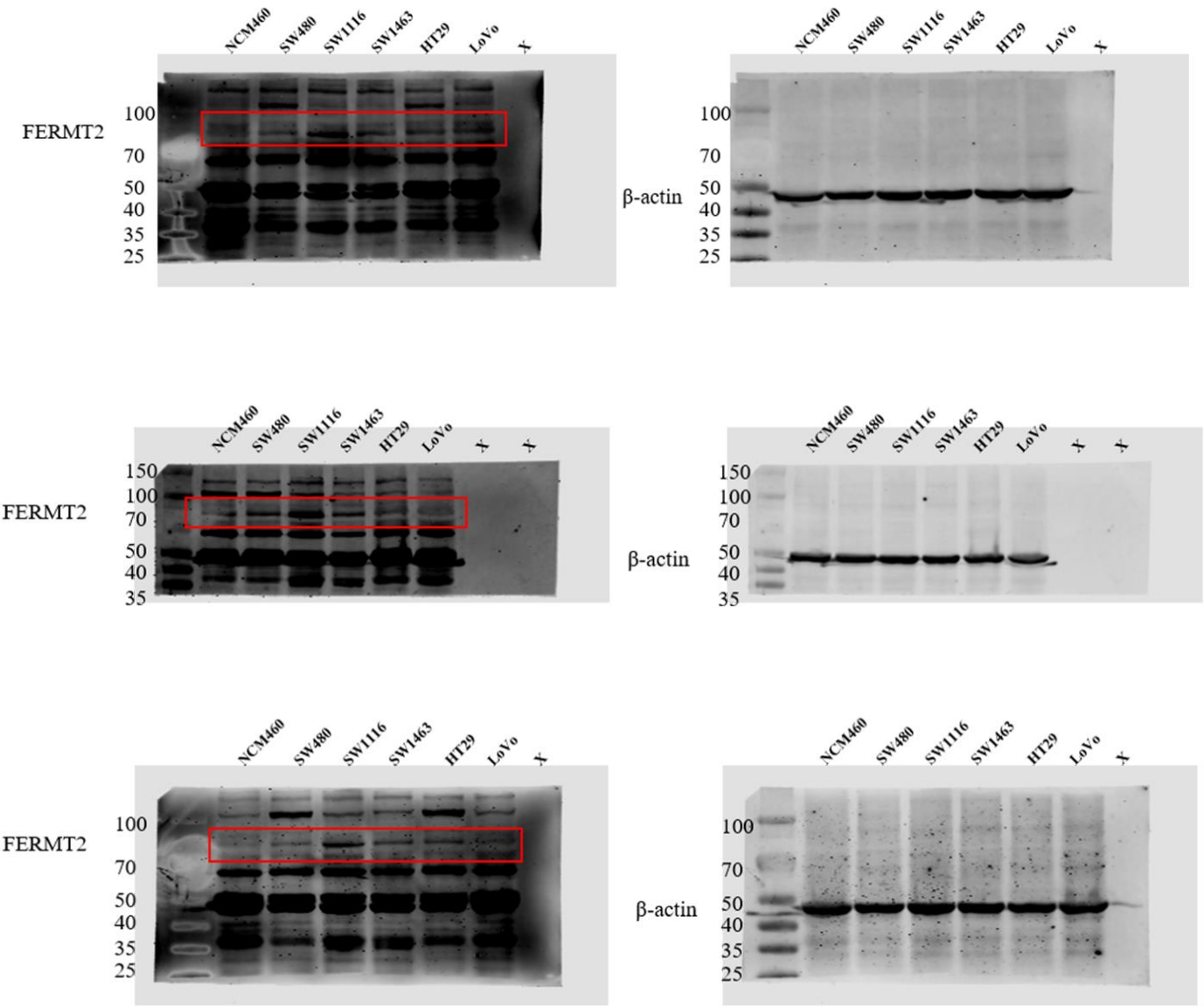

**Fig 5D**

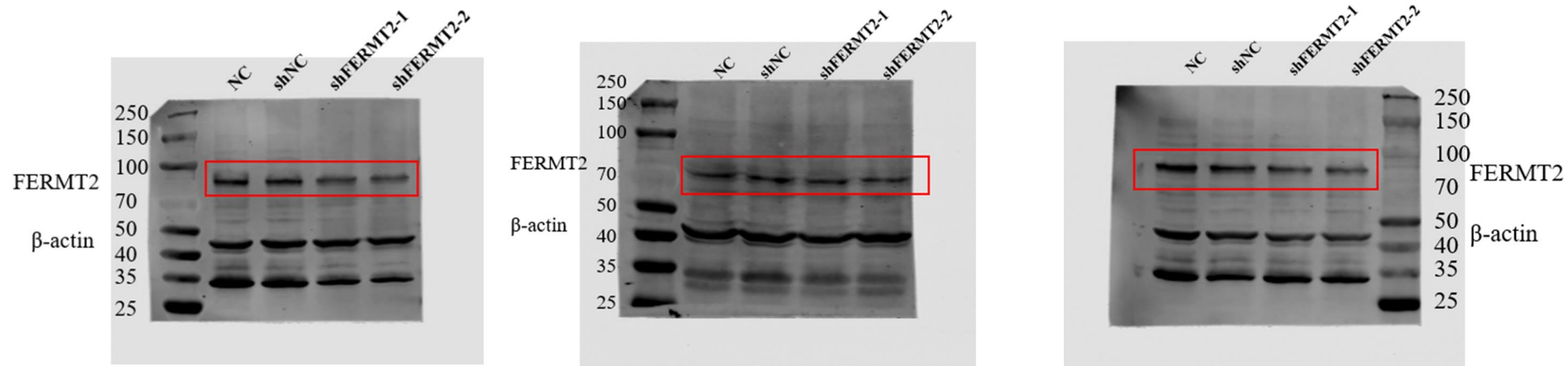

**Fig 5F**

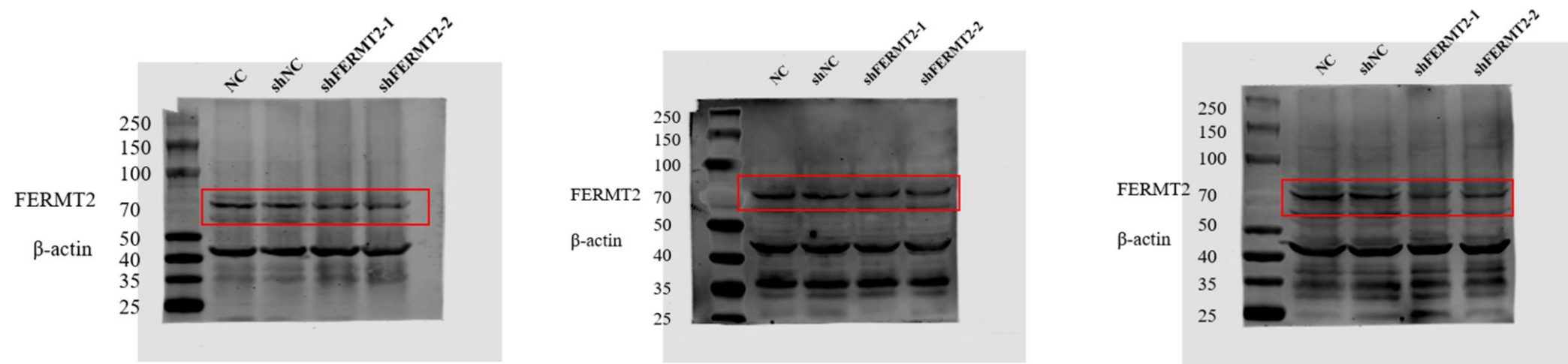

**Fig 6E**

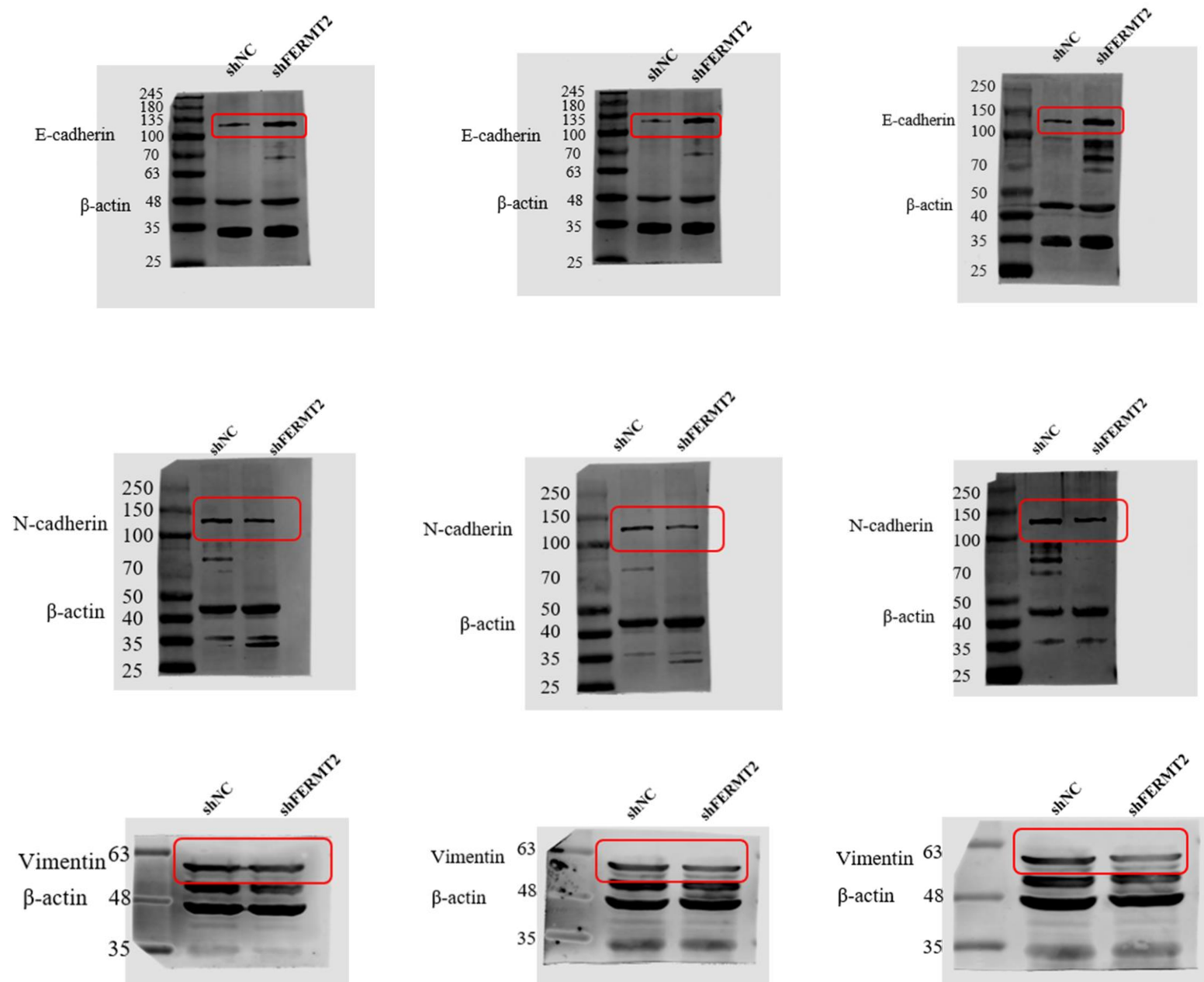

**Fig 6G**

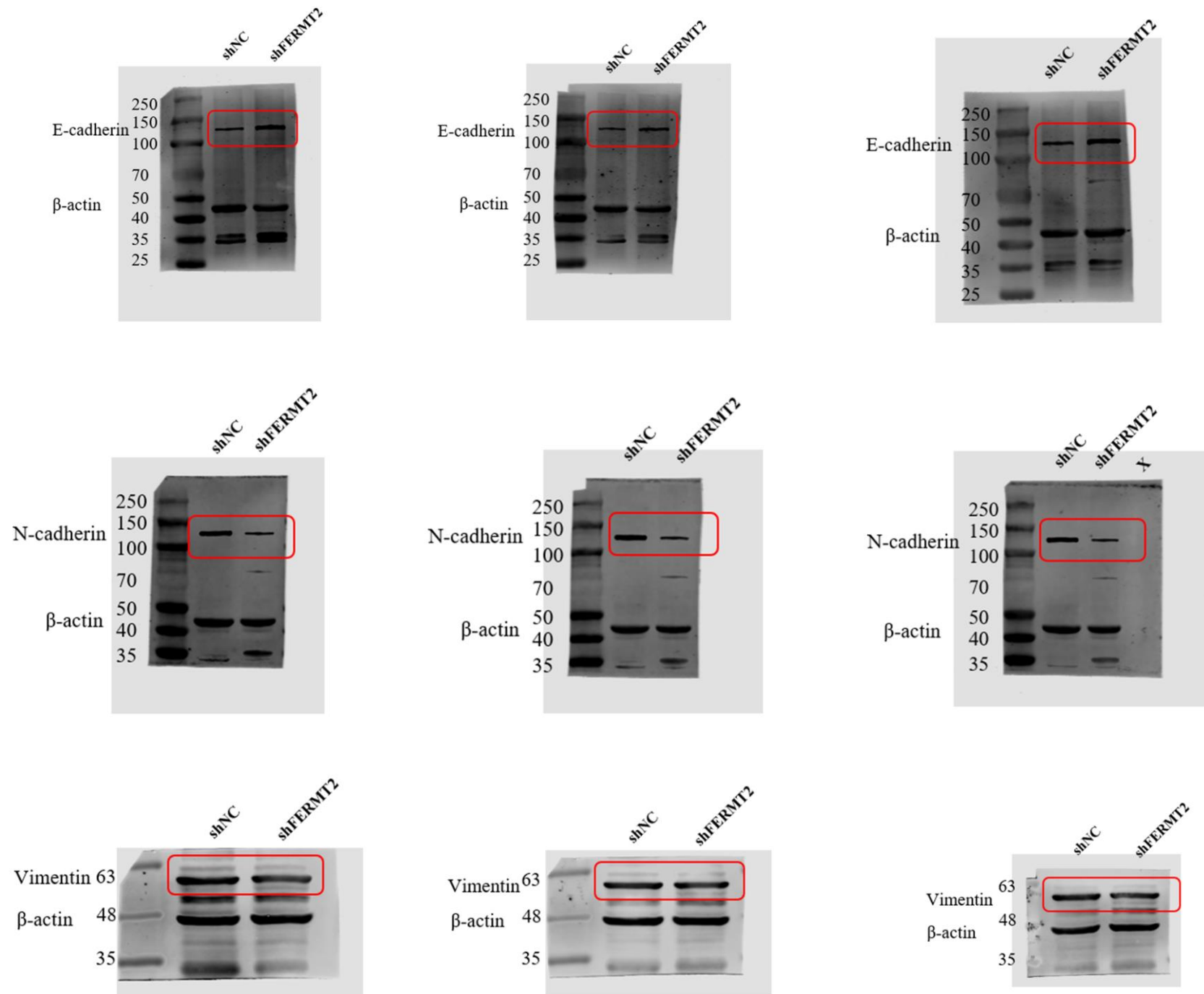

**Fig 7B**

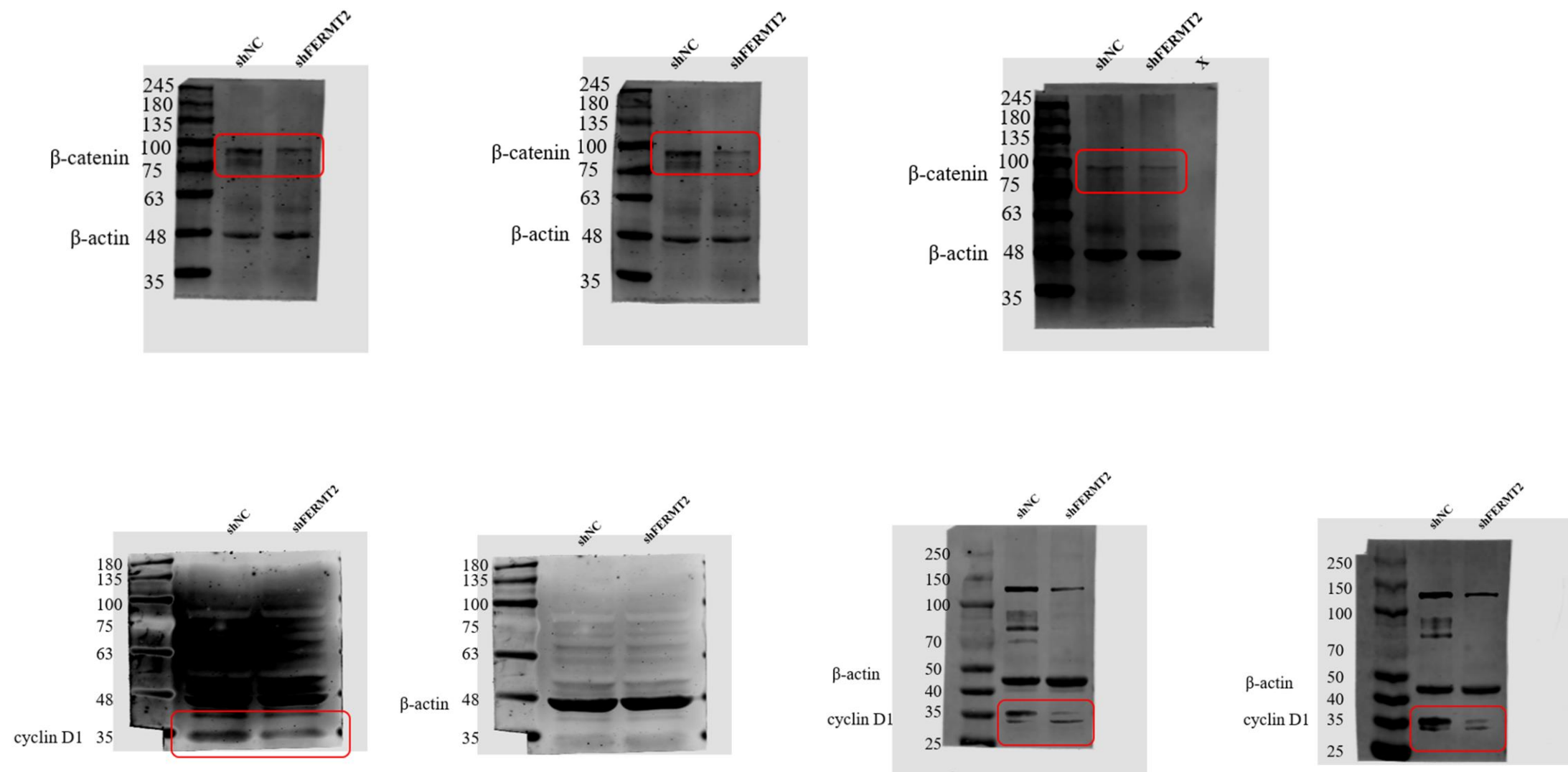

**Fig 7C**

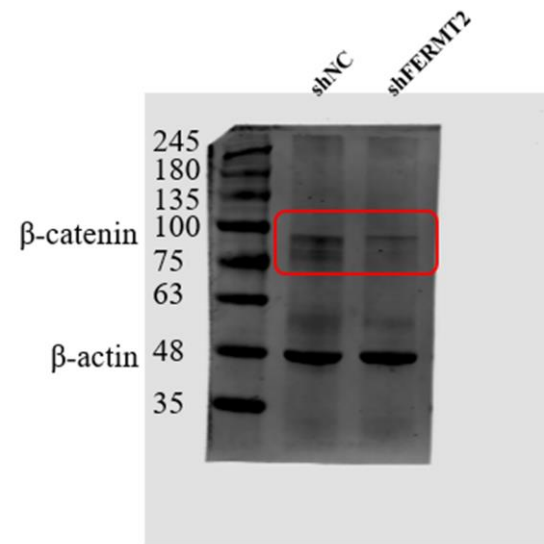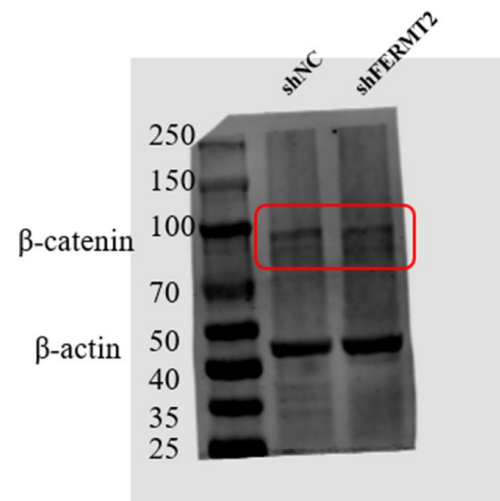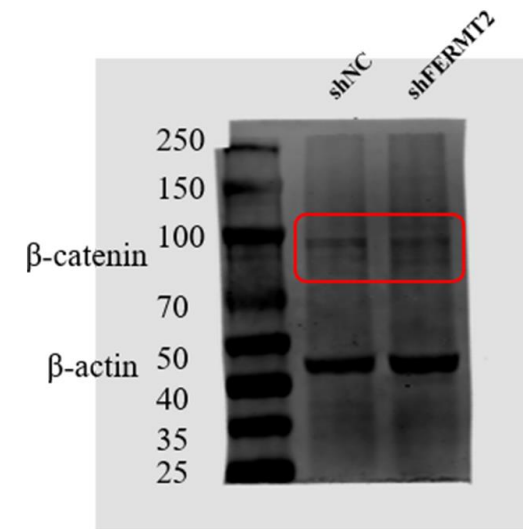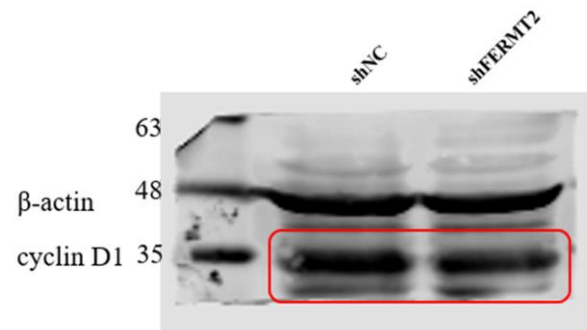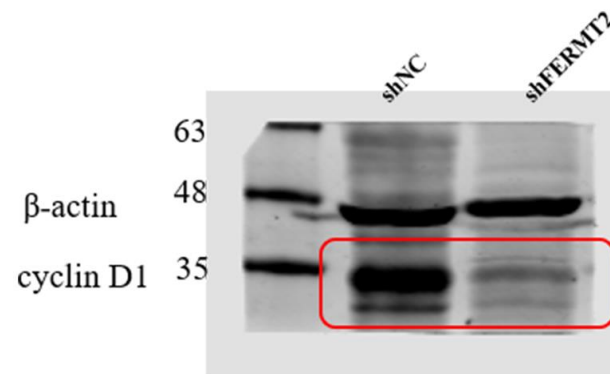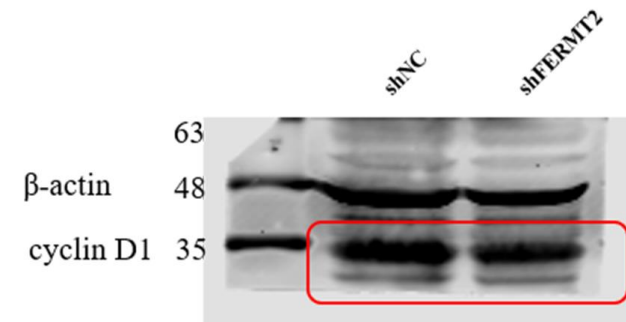

**Fig 7D**

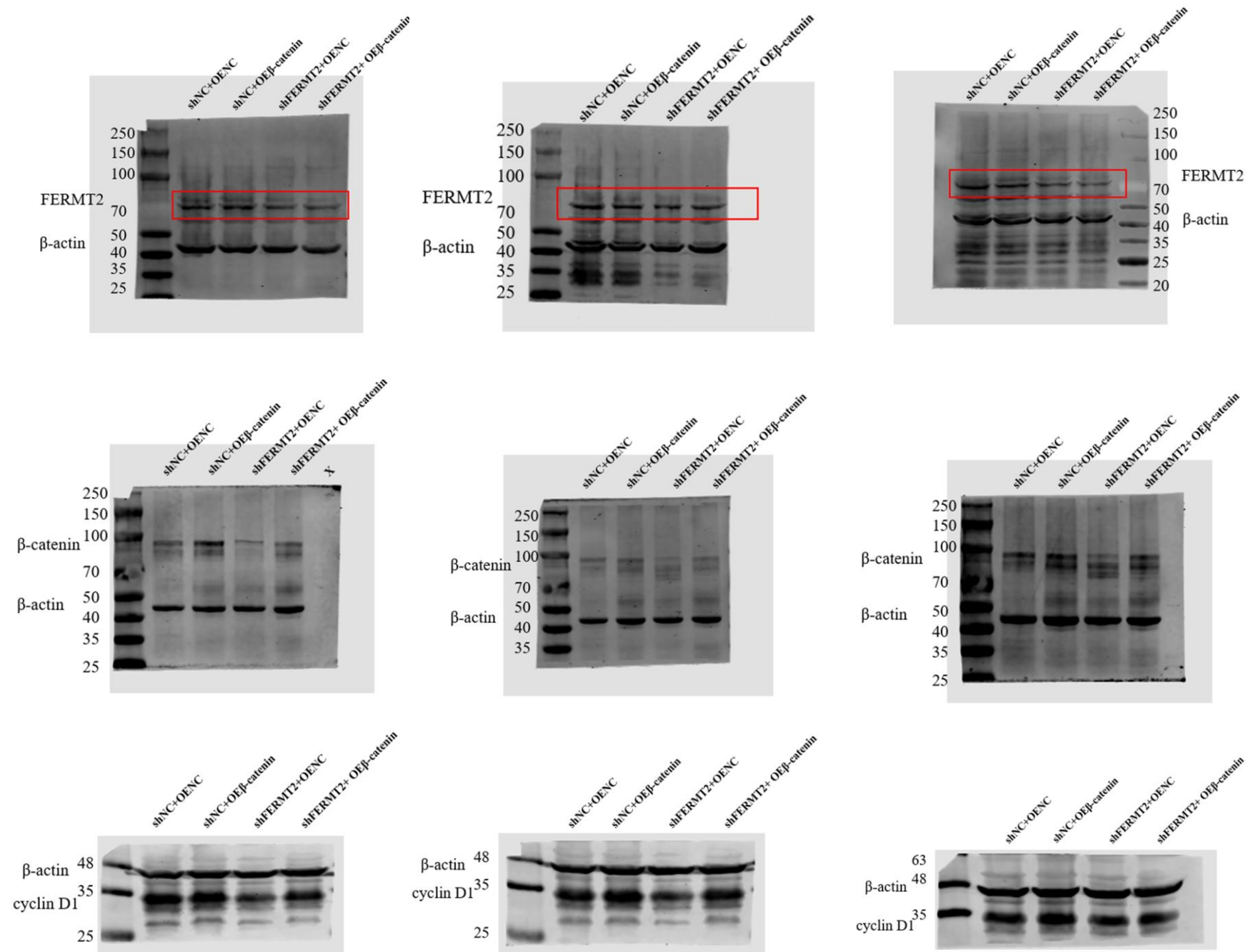

**Fig 7E**

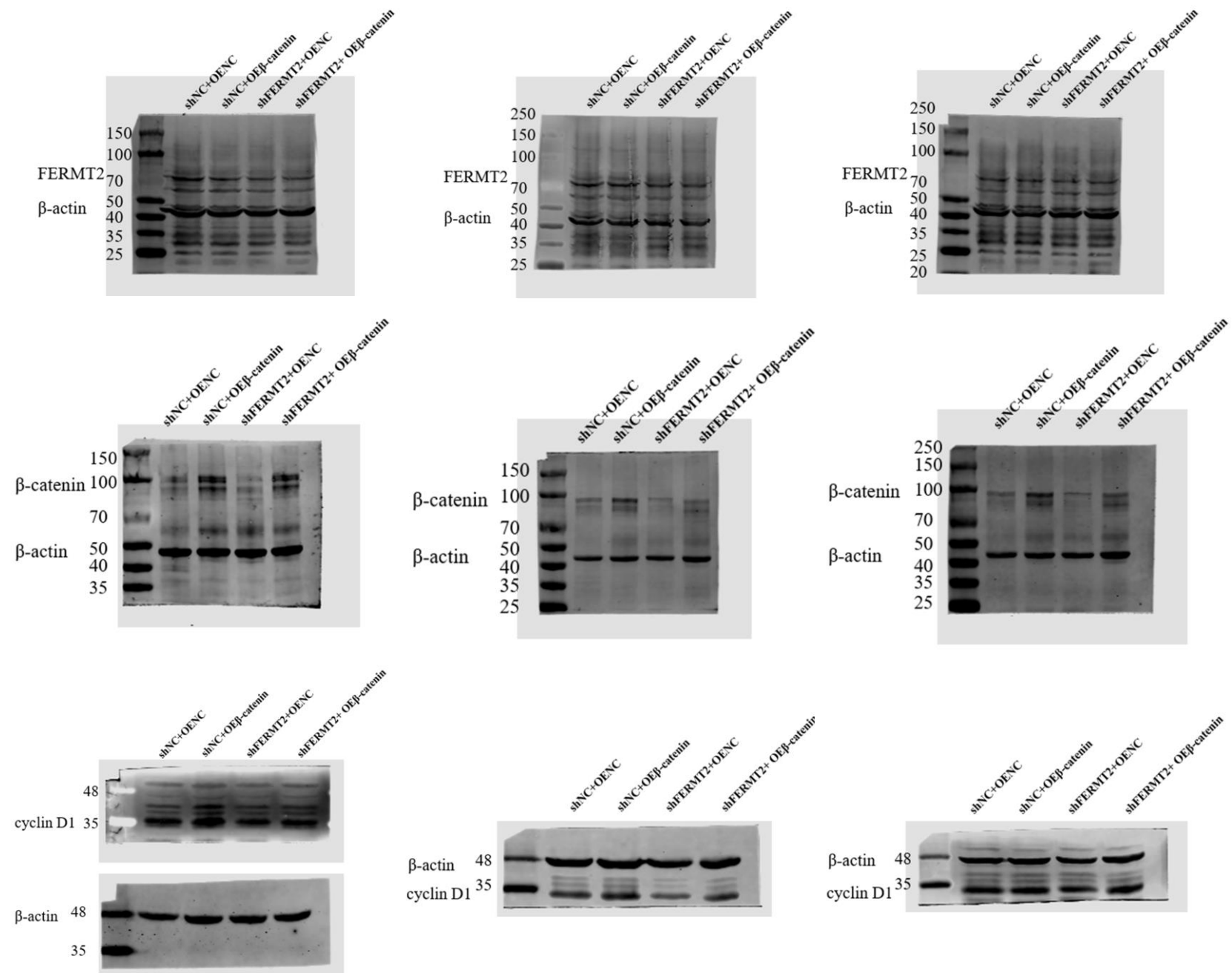

**Fig 8B**

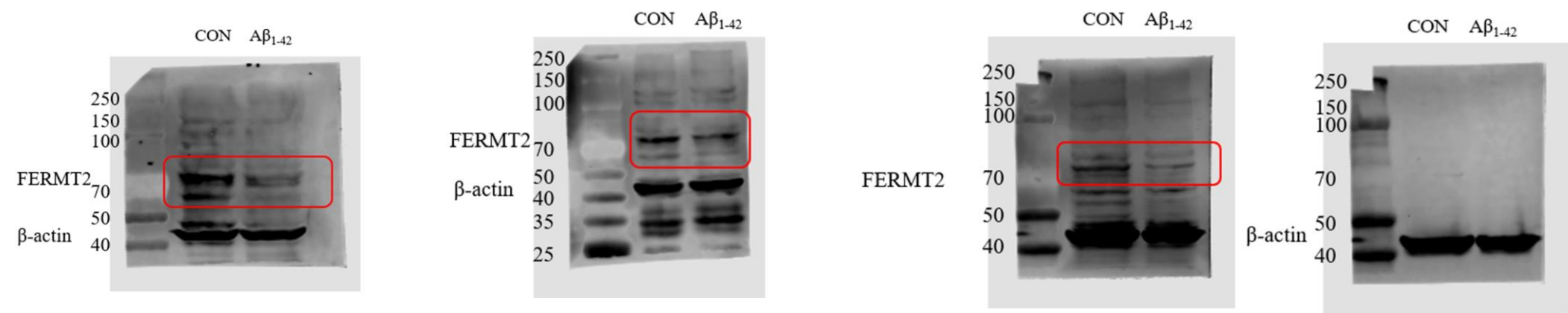

**Fig 8F**

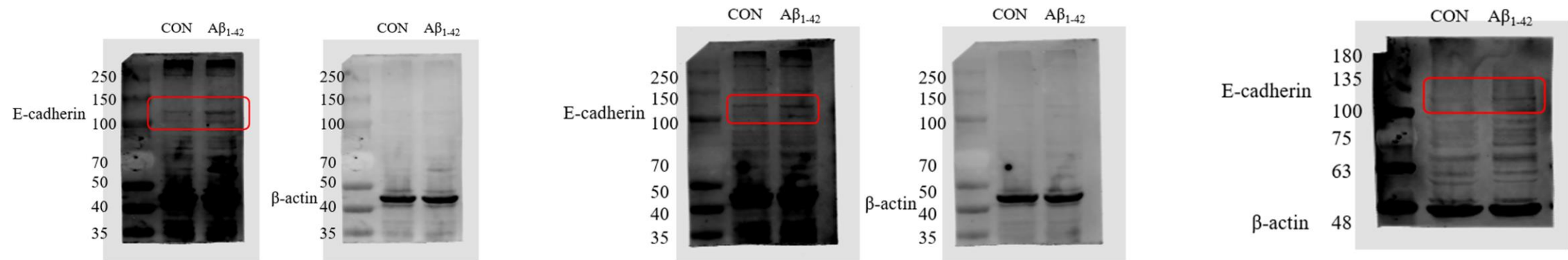

Fig 8F

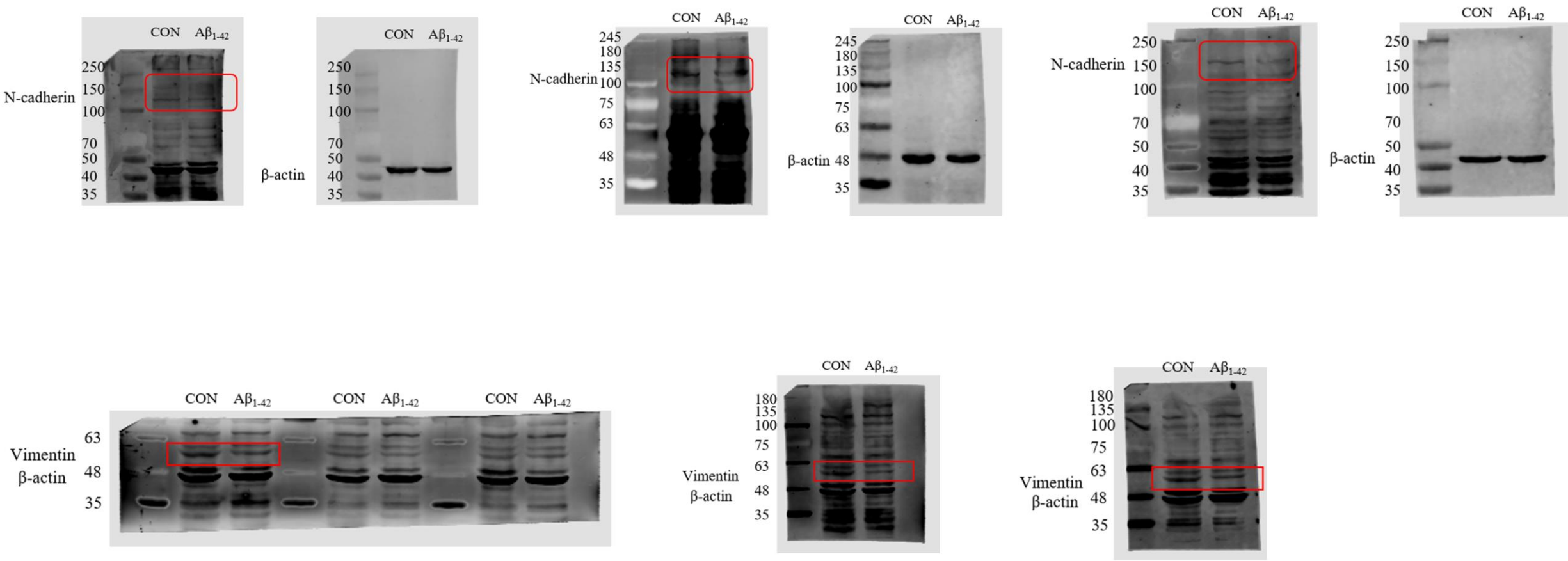

Fig 8H

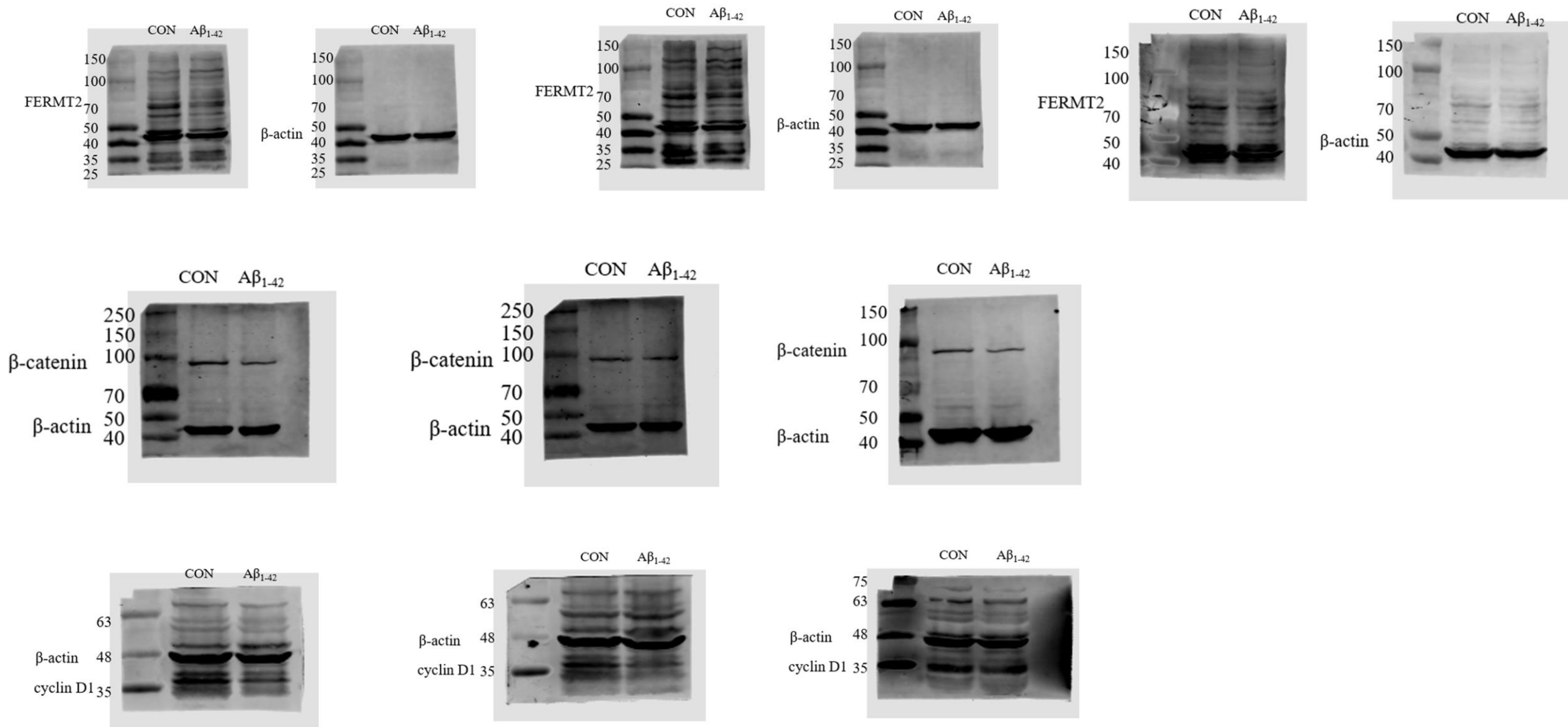

**Fig 9A**

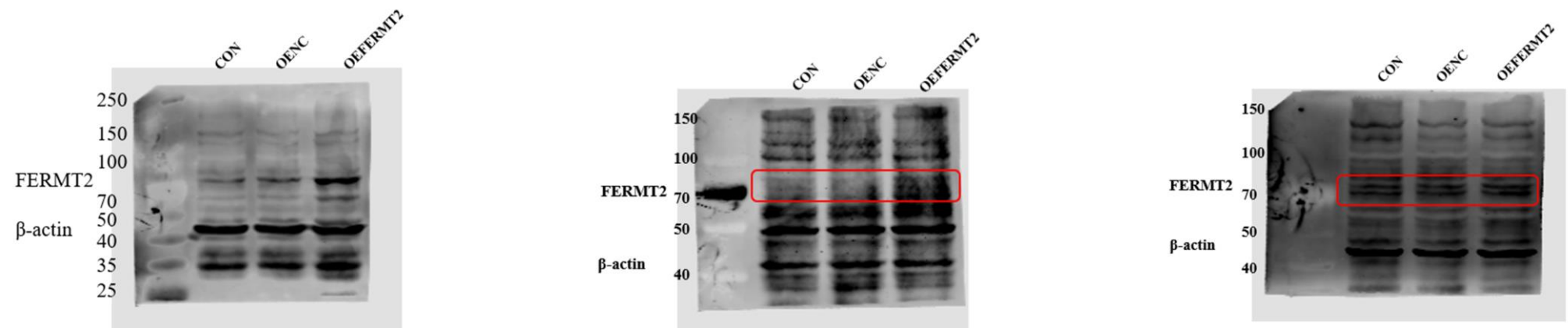

**Fig 9B**

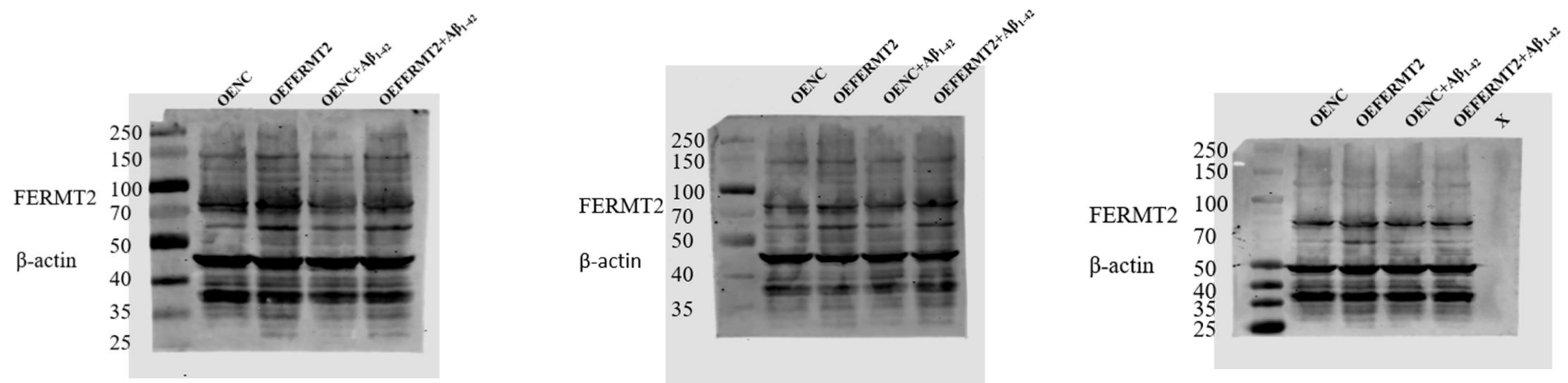

Fig 10A

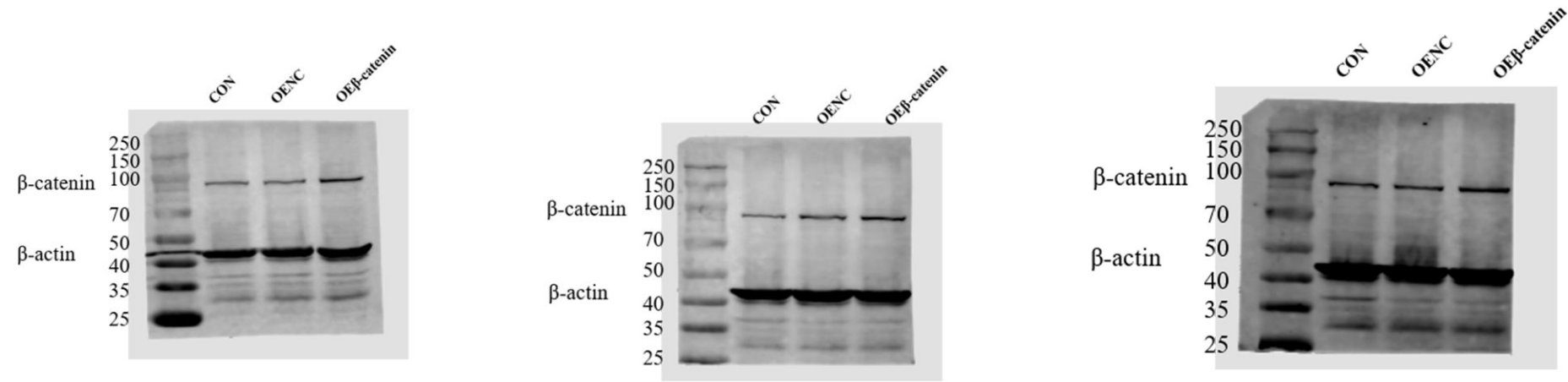

Fig 10E

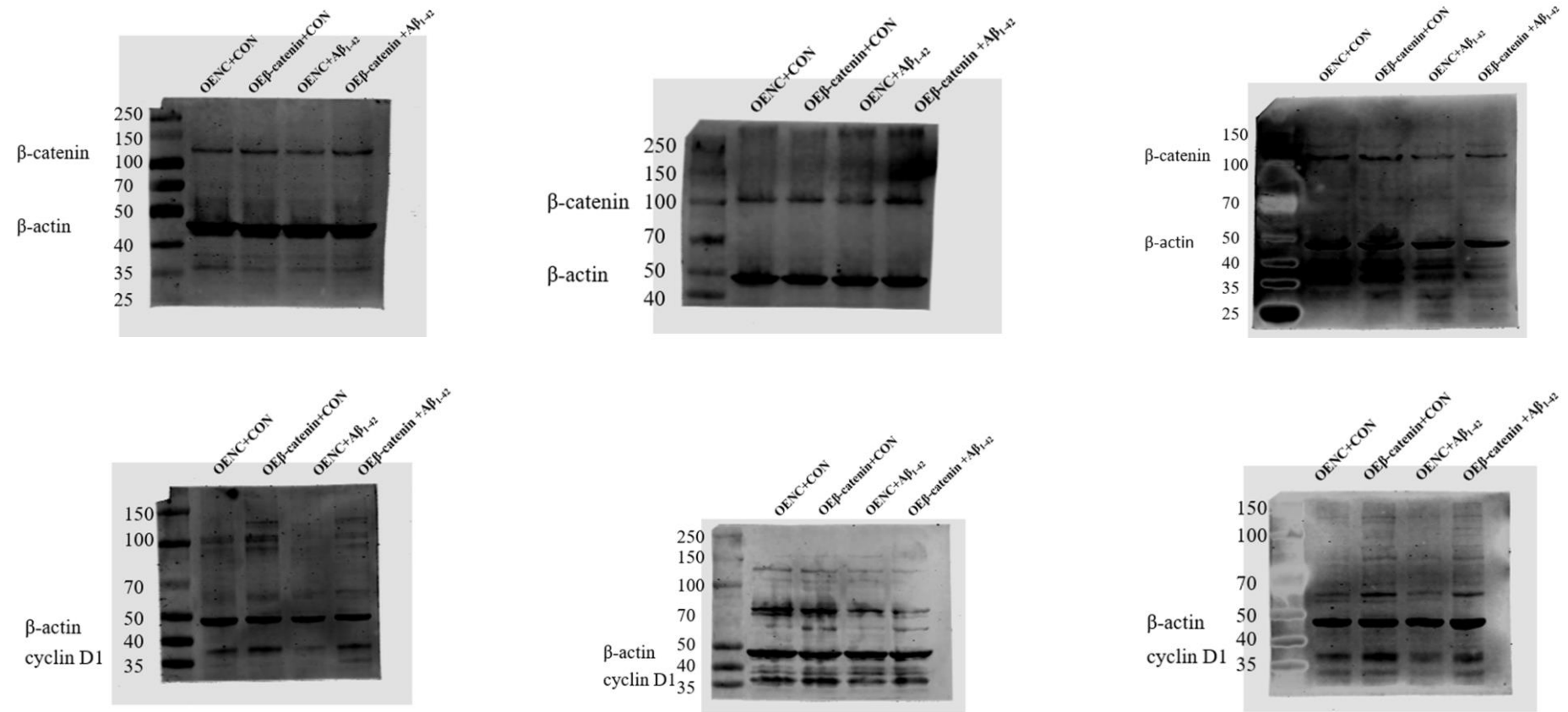

Supplement: S1 Raw images — (PDF) [file pone.0278774.s006.pdf]
