# Supplementary material for: Alzheimer’s risk factor FERMT2 promotes the progression of colorectal carcinoma via Wnt/β-catenin signaling pathway and contributes to the negative correlation between Alzheimer and cancer
Source: PLoS One. 2022 Dec 8;17(12):e0278774. doi: 10.1371/journal.pone.0278774 (PMC9731493; doi:10.1371/journal.pone.0278774)
Supplement: S1 File — (DOCX) [file pone.0278774.s005.docx]

1. A link for raw data of western blot:

https://www.jianguoyun.com/p/DVif3s4Q-4X0ChjW99QEIAA

2. A link for raw data of the manuscript:

https://www.jianguoyun.com/p/Dca-pyIQgtLkChihgs4EIAA
